# Profiling of Humoral Response to Influenza A(H1N1)pdm09 Infection and Vaccination Measured by a Protein Microarray in Persons with and without History of Seasonal Vaccination

**DOI:** 10.1371/journal.pone.0054890

**Published:** 2013-01-24

**Authors:** Elisabeth G. W. Huijskens, Johan Reimerink, Paul G. H. Mulder, Janko van Beek, Adam Meijer, Erwin de Bruin, Ingrid Friesema, Menno D. de Jong, Guus F. Rimmelzwaan, Marcel F. Peeters, John W. A. Rossen, Marion Koopmans

**Affiliations:** 1 Laboratory of Medical Microbiology and Immunology, St. Elisabeth Hospital, Tilburg, The Netherlands; 2 National Institute of Public Health and the Environment, RIVM, Bilthoven, The Netherlands; 3 Amphia Academy, Amphia Hospital, Breda, The Netherlands; 4 Department of Medical Microbiology, Academic Medical Center, University of Amsterdam, Amsterdam, The Netherlands; 5 Department of Virology, Erasmus Medical Center, Rotterdam, The Netherlands; 6 Department of Medical Microbiology, University of Groningen, University Medical Center Groningen, Groningen, The Netherlands; The Ohio State University, United States of America

## Abstract

**Background:**

The influence of prior seasonal influenza vaccination on the antibody response produced by natural infection or vaccination is not well understood.

**Methods:**

We compared the profiles of antibody responses of 32 naturally infected subjects and 98 subjects vaccinated with a 2009 influenza A(H1N1) monovalent MF59-adjuvanted vaccine (Focetria®, Novartis), with and without a history of seasonal influenza vaccination. Antibodies were measured by hemagglutination inhibition (HI) assay for influenza A(H1N1)pdm09 and by protein microarray (PA) using the HA1 subunit for seven recent and historic H1, H2 and H3 influenza viruses, and three avian influenza viruses. Serum samples for the infection group were taken at the moment of collection of the diagnostic sample, 10 days and 30 days after onset of influenza symptoms. For the vaccination group, samples were drawn at baseline, 3 weeks after the first vaccination and 5 weeks after the second vaccination.

**Results:**

We showed that subjects with a history of seasonal vaccination generally exhibited higher baseline titers for the various HA1 antigens than subjects without a seasonal vaccination history. Infection and pandemic influenza vaccination responses in persons with a history of seasonal vaccination were skewed towards historic antigens.

**Conclusions:**

Seasonal vaccination is of significant influence on the antibody response to subsequent infection and vaccination, and further research is needed to understand the effect of annual vaccination on protective immunity.

## Introduction

The first influenza pandemic of the 21st century was caused by a novel influenza A(H1N1) virus, which was a complex reassortant virus containing genes from avian, human, and swine influenza viruses. [1] Hemagglutinin (HA) rapidly and continuously accumulates mutations to escape recognition by virus-specific antibodies. To date, epidemics and pandemics of influenza in humans have been restricted to viruses with subtype H1, H2, and H3 surface HAs, combined with neuraminidase proteins (NA) of subtypes N1 and N2. However, sporadic zoonotic infections with avian influenza viruses of subtypes H5, H7 and H9 have been documented. [2] The potential diversity of influenza viruses is larger, as sixteen subtypes of HA and 9 subtypes of NA have been identified in wild waterfowl, that constitute a reservoir for influenza viruses. [3] The occasional zoonotic transmissions, and the opportunity for human adaptation of animal influenza viruses through reassortment or adaptation, constitute a continuous pandemic threat, as illustrated by the recent pandemic in 2009. Impact of such a new introduction is determined in part by the level of pre-existing immunity in the population. Natural influenza virus infection elicits a protective immune response, mediated primarily through neutralizing antibodies directed to host-cell binding domains on the surface proteins of the infecting strain and antigenically related viruses. An important question related to the emergence of new influenza viruses, however, is the degree of antigenic mismatch that can be tolerated before virus-neutralising antibodies are no longer capable of inhibiting infection. Also, the role of antibodies to other epitopes is poorly understood. Recently, human monoclonal antibodies against highly conserved influenza virus epitopes in the stalk region were discovered with broad neutralizing activity against a wide spectrum of influenza subtypes. [4], [5] Similarly, low level cross-reactive antibodies that bind to the globular head (HA1) have been found in some individuals (Baas et al., submitted for publication). An important question is whether the presence of such broad non-neutralising antibodies may somehow influence infection. During the recent pandemic, this discussion was further triggered because of the observed discrepancy between the population immunity estimates based on serology and the observed impact: cross-neutralizing antibodies were found in persons exposed to historic influenza A(H1N1) strains that were circulating prior to the emergence of the pandemic influenza H2N2 strain in 1956/57. [6] Nevertheless, only a small fraction of persons older than 20 years of age were infected during the first pandemic wave, suggesting other factors influencing population susceptibility. [7] Wrammert et al. [8] identified broadly cross-reactive neutralizing antibodies induced by infection between the influenza A(H1N1)pdm09, recent seasonal influenza A(H1N1) strains, as well as influenza A(H1N1)1918, and avian influenza viruses of subtypes H5N1. Others showed that seasonal vaccination can induce heterosubtypic neutralizing antibodies as well. [9], [10] Somewhat contrasting with this is the observation that a history of seasonal vaccination can lead to lower levels and shorter duration of the strain-specific antibody responses upon heterologous infection. [11], [12].

These findings show that the exposure history of individuals needs to be considered in order to better understand the role of antibodies in susceptibility to infection,. A commonly used measure for determination of protective antibodies induced by influenza virus infection and vaccination is the hemagglutination inhibition (HI) assay, where a HI titer ≥40 has been associated with 50% protection against influenza virus infection in susceptible populations. [13] HI assays, however, lack reproducibility between laboratories, for example due to inter-observer variability in visual read-outs of the agglutination titer and the nature and quality of the erythrocytes that are used in the assay. In addition, a practical limitation is the need for high amounts of virus and serum when antibodies to multiple strains need to be determined, and a biosafety level II and III working environment. [13], [14] Testing for antibodies by micro-neutralization assay has similar disadvantages, and is not widely available, thereby limiting their use for comparative studies. ELISA assays have suffered from lack of specificity, due to broad cross reactivity when HA antigens are used. Therefore, we explored alternatives for HI antibody testing, and developed a protein microarray based assay to measure antibodies to the HA1 subunit from a wide range of viruses, including seven recent and historic seasonal H1, H2 and H3 influenza viruses, the A(H1N1)pdm09 influenza virus, and three avian influenza viruses. [15] Use of this assay revealed substantial diversity in the antibody profile of individuals, depending on age, but also on exposure history and on individual host responses. In this study, we compared the profile of antibody responses elicited by natural infection, and vaccination for influenza A(H1N1)pdm09 in healthy adults with and without a history of seasonal influenza vaccination using the protein microarray.

## Materials and Methods

### Ethics Statement

Both studies were approved by the appropriate institutional review boards; the Medical Ethical Review Committee of the St. Elisabeth Hospital, Tilburg, The Netherlands and the Medical Ethical Review Committee of the University Medical Centre, Utrecht. Written informed consent was obtained from the participant or parents/guardians in case of the children.

### Subjects

#### Vaccination group

We conducted a prospective, longitudinal study from November 2009 through June 2010 at the St. Elisabeth and TweeSteden Hospital, Tilburg, The Netherlands. [12] Healthcare workers (≥18 years; if pregnant only after 13 weeks of pregnancy) of both hospitals were eligible for inclusion. Serum samples were collected prior to the first vaccination with a 2009 influenza A(H1N1) monovalent MF59-adjuvanted vaccine (Focetria®, Novartis), before the second vaccination (three weeks later) and before the vaccination with trivalent seasonal influenza vaccine (5 weeks after the second vaccination). Demographic characteristics (age and sex), seasonal influenza vaccination status, and comorbidity were collected by means of a short questionnaire. Two subgroups were made: a group that never received seasonal vaccination and a group that received seasonal influenza vaccination annually.

#### Infection group

We used serum samples from a national pandemic influenza cohort study. [16] Patients and some of their household contacts had been diagnosed with influenza during the active case finding activities instituted in the early phase of the pandemic. Persons testing positive for influenza A(H1N1)pdm09 by RT-PCR testing of a throat/nose swab were contacted and asked if they were willing to participate in a national cohort study. We obtained three samples; the first sample taken between 0 - 5 days after onset of influenza symptoms, the 2nd at 10 days after onset, and the third sample at around 30 days. We used data on age, sex, date of onset of illness, and seasonal influenza vaccination status. Two subgroups were made: a group not receiving regular vaccination and a group that was regularly vaccinated.

### Antibody-titer Determination by Hemagglutination Inhibition Assay (HI)

Virus specific antibodies were measured by HI assay, using egg-grown A/California/7/2009 A(H1N1) pandemic virus, and fresh red blood cells of turkeys in Alsever’s solution (Biotrading, The Netherlands), according to standard methods. [12] The HI titer was the reciprocal of the highest dilution of serum that inhibited virus induced hemagglutination. Titers below the detection limit of 10 were assigned to a value of 5, and 1280 was the end point titration and also the highest dilution tested. Titers were calibrated against a candidate International Standard for antibody-titers to influenza A(H1N1)pdm09 virus. [17].

### Antibody Determination by Protein Array (PA)

Antibody titers were determined by PA as previously described. [15] Briefly serum samples were tested in 2 fold serial dilutions from 1∶20 to 1∶2560 on nitrocellulose slides pre-coated with a selection of recombinant monomeric HA1 proteins (**Table 1**). Inter-assay variability was monitored by testing dilutions of a candidate International Standard for antibody-titers to influenza A(H1N1)pdm09 virus. [17] Trays with a H1 titer for the International Standard deviating more than one titer step from the GMT of all standards in the particular run were rejected. Microarray slides were scanned using a ScanArray Gx Plus microarray scanner (PerkinElmer) and median spot fluorescence intensity was determined by using ScanArray Express (version 4.0) software (PerkinElmer). Titers were calculated from the inflection point of the titration curve as described. [15] Specificity of reactivity was determined using subtype-specific rabbit antisera, sera from persons of different age-groups with exposure to different seasonal influenza viruses, and supernatants from cloned human memory B cells. Full details of the array validation have been published elsewhere.( [15], Baas et al., submitted).

**Table 1:** HA1 antigens used for the microarray.

Name	Strain	Subtype
H1-1999	A/New Caledonia/20/99	H1N1
H1-2007	A/Brisbane/59/2007	H1N1
H1-1933	A/WS/33	H1N1
H1-2009	A/California/6/2009	H1N1
H1-1918	A/South Caroline/1/18	H1N1
H2-1957	A/Canada/720/05	H2N2
H3-2003	A/Wyoming/3/03	H3N2
H3-2007	A/Brisbane/10/2007	H3N2
H5-2004	A/Vietnam/1194/2004	H5N1
H7-2003	A/Chicken/Netherlands/1/03	H7N7
H9-1999	A/Guinea fowl/Hong Kong/WF10/99	H9N2

### Statistical Analyses

Differences in age and gender between the vaccination and infection group were tested using the Mann-Whitney and chi-square test, respectively. Antibody levels were analyzed for both groups against timing of sampling, age, gender, and vaccination history, using linear mixed modeling after log-transformation. Separate analyses were performed in the vaccination and infection group. Also the interaction between time (three time points) and seasonal vaccination (yes/no) was entered in the model if significant. No structure was imposed on the residual (co)variances of the three repeated titer measurements. Estimated coefficients and their 95% confidence limits were back-transformed to be interpretable as geometric mean titers (GMT) and multiplicative change factors between those GMTs.

A titer rise was defined as a 4-fold or greater increase in antibody titer between the first sample (with a minimum titer of 40 for PA) and follow-up samples from the same individual. Seroconversion was defined as an increase from below to above a titer of 40.

## Results

### Comparison of Vaccination- and Natural Infection-group

A total of 130 subjects were included, with 98 persons in the vaccination group and 32 in the natural infection-group. Details of the study groups are shown in **Table 2**. The median age of the vaccination group was 49 years (range, 25 to 66), 35 (35.7%) were men; 40 subjects (40.8%) had received annual seasonal influenza vaccination. The naturally infected patients were younger (p<0.0005) and were more often male (p 0.040) than in the vaccination group: the median age was 30 years (range, 12 to 66), 18 (56.3%) were men; 15 subjects (46.9%) had regularly been vaccinated against seasonal influenza virus.

**Table 2:** Demographic characteristics of the subjects.

Characteristics	All subjects	Not yearly vaccinatedwith seasonal vaccine	Yearly vaccinated with seasonalvaccine	Natural infection withH1N1(2009) without earlierseasonal vaccine	Natural infection withH1N1(2009) with earlierseasonal vaccine
	*n = 130*	*n = 58*	*n = 40*	*n = 17*	*n = 15*
**Age - yrs.**					
Median	48	48	51	31	29
Range	12–66	28–61	25–66	14–60	12–66
**Sex – no. (%)**					
Male	53 (40.8)	20 (34.5)	15 (37.5)	10 (58.8)	8 (53.3)
Female	77 (59.2)	38 (65.5)	25 (62.5)	7 (41.2)	7 (46.7)
**Vaccinated since (%)**					
Before 2000			15 (37.5)		Nk*
2000–2004			20 (50.0)		Nk*
After 2004			5 (12.5)		Nk*

* Not known.

### Kinetics of Antibody Response to Influenza A(H1N1)pdm09

**Figure 1:**
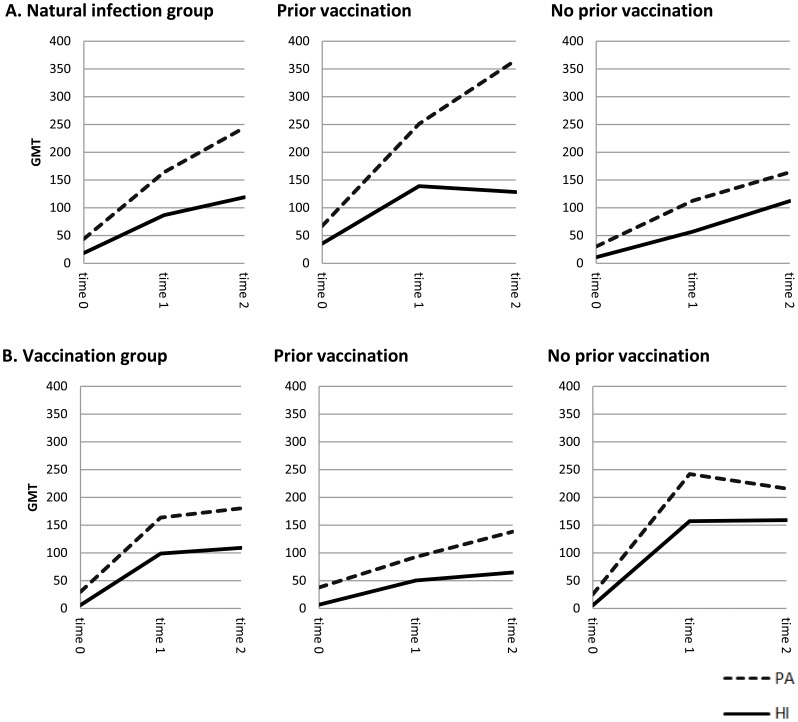
HI versus PA for influenza A(H1N1)pdm09 in the natural infection-group and in the vaccination group. A. GMTs of influenza A(H1N1)pdm09 for HI versus PA in the natural infection-group, with and without prior seasonal vaccination at time point 0 represents baseline, time point 1 represents 10 days after day of onset of influenza symptoms and time point 2 represents 30 days after day of onset of influenza symptoms. **B.** GMTs of influenza A(H1N1)pdm09 for HI versus PA in the vaccination group, with and without prior seasonal influenza vaccination at time point 0 represents baseline, time point 1 represents 3 weeks after the first vaccination and before the second vaccination and time point 2 represents 5 weeks after the second vaccination. Y axis denotes average GMT (adjusted for gender and age).

Trends in GMTs at baseline and at the different time points are shown for both groups in **Figure 1**. Seventy-two of the 98 vaccinees and 22 of the 32 infected patients showed a titer increase or seroconversion for the homologous antigen as measured by PA, and 91 of the 98 vaccinees and 27 of the 32 infected patients as measured by HI.

When comparing titers against the homologous antigen A(H1N1)pdm09, GMTs measured by PA were higher than those measured by HI at all time points in both groups, with no obvious differences between vaccinees and patients. However, when stratifying the data according to seasonal vaccination history, a difference in antibody responses was observed between both groups. Naturally infected persons with a history of seasonal vaccination showed a stronger antibody response to A(H1N1)pdm09 by both HI and PA than persons without a history of seasonal vaccination. This contrasted with opposite results in persons who received the pandemic vaccine: vaccinees with a history of seasonal influenza vaccination showed a less pronounced response by both methods than persons who were never vaccinated. Similar to observations for the whole groups, the magnitude of responses measured by the two techniques also differed, with highest GMTs measured by PA. The antibody responses measured by both methods were co-linear, except for the naturally infected persons with a history of vaccination: here, the curve for the response measured by PA was steeper, suggesting a disproportionate increase in non-HI antibodies in this group.

### Antibody Expression Profiles

To gain a more detailed understanding of the quality and composition of the antibody response, we profiled sera against 7 antigens using the PA. The expression profile of the different antigens in response to natural infection and vaccination against influenza A(H1N1)pdm09 was analyzed using linear mixed modeling, with adjustments made for gender and age. A ratio was calculated to indicate the increment between groups with and without seasonal vaccination.

#### Antibody profile in response to influenza A(H1N1)pdm09 virus infection with and without seasonal influenza vaccination

**Figure 2:**
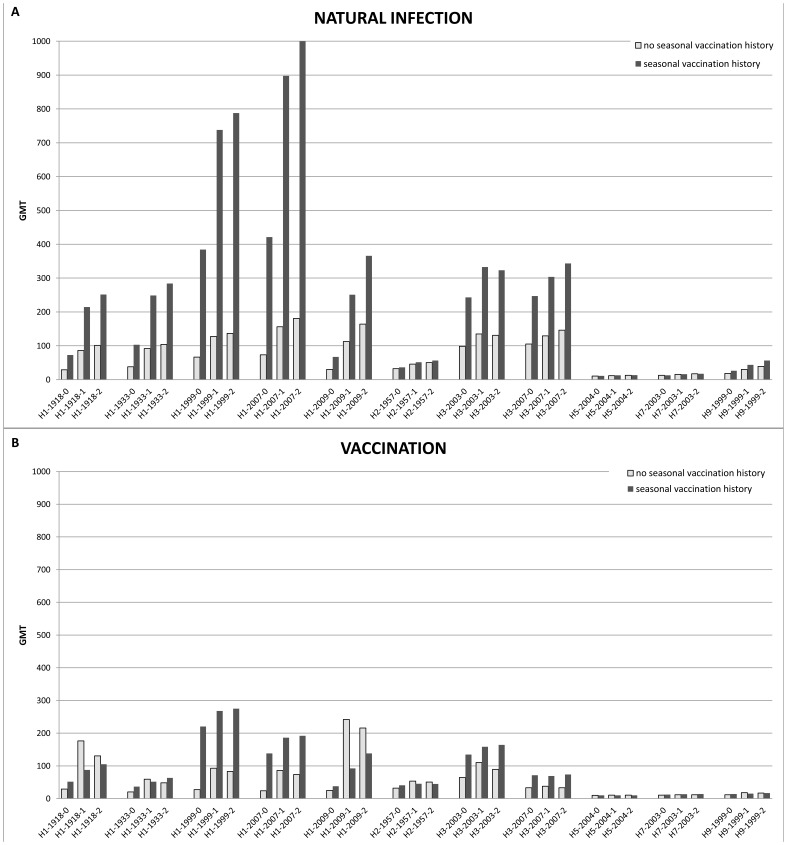
Geometric mean titers (GMT) at time point 1 and 2 of the natural infection-group and in the vaccination group with and without former seasonal influenza vaccination. A. GMTs for the various influenza HA1 antigens in patients infected with pandemic influenza H1 2009, with and without a history of seasonal vaccination at time point 1 (10 days after day of onset of influenza symptoms) and time point 2 (30 days after day of onset of influenza symptoms). **B.** GMTs for the various influenza HA1 antigens in subjects vaccinated with inactivated MF-59 adjuvated pandemic Influenza A virus (H1N1) 2009, with and without a history of seasonal influenza vaccination at time point 0, 1 (3 weeks after the first vaccination and before the second vaccination) and time point 2 (5 weeks after the second vaccination). Y axis denotes average GMT (adjusted for gender and age).

When comparing change in titer compared to baseline in the natural infection-group, the greatest increase (fold change) was observed for the homologous antigen, followed by 1918 and the other H1 antigens. Smaller but significant rises in antibody titer were observed for all other antigens except H5-2004. Subjects with a history of seasonal influenza virus vaccination showed a significantly higher baseline titer for the historic and recent H1 and H3 influenza antigens, but not for H2 and the avian influenza virus antigens (**Figure 2**). The natural infection-group had a greater increase in titer for all antigens than the vaccination group, although the response in the persons with seasonal vaccination history was skewed towards seasonal influenza antigens H1-1999 and H1-2007, for which most significant differences in response were observed. No significant interaction between time and former seasonal vaccination was found for any of the antigens in this infection group, adjusted for age and gender. Absence of this above mentioned interaction coincides with a model where the time courses of the titers in both vaccination history subgroups are parallel. (Described in Table S1).

#### Antibody profile in response to vaccination against influenza A(H1N1)pdm09 with and without seasonal influenza vaccination

In persons without a history of seasonal vaccination, the magnitude of the antibody response (fold change) was highest for the homologous and the H1-1918 antigens. Smaller rises in antibody titers were observed for all other seasonal H1 viruses and to a lesser extent for H2 and H3. In persons with a history of annual seasonal vaccination, the GMT at baseline was higher than those without previous vaccination, but the magnitude of response was much lower, and narrower, with seroconversion or significant increase in titer largely limited to the H1-2009 antigen. In persons without a history of seasonal vaccination, peak responses were measured already at the time of the booster vaccination with the pandemic vaccine, whereas this was not the case for persons with a history of seasonal influenza vaccination: here, the maximum change in titer was lower, and continued to increase until the third time point of sampling, after the second vaccine dose was given as seen in **Figure 2**. (Further described in Table S2).

## Discussion

In the present study, we compared antibody profiles in response to infection and vaccination with influenza A(H1N1)pdm09 with a PA. This technology was developed to enable comparative studies using standardized assay format to reduce the problem of variability and potentially interlaboratory differences in results of HI and microneutralization testing by obviating the need for use of biological reagents that are difficult to standardize, such as red blood cells (for HI assays) or cells (for virus neutralization assays). [15] We demonstrated that subjects with a history of seasonal vaccination generally exhibited higher baseline titers for the various HA antigens than subjects without such history. We also show that the response differs according to the type of exposure: following natural infection, a strong homologous response, and weaker but significant increases in antibody titers to different HA1 antigens are seen, whereas the response following vaccination was more restricted. These results need to be interpreted with caution, because the mean age of the persons in the infection group was lower, which in part could explain a more vigorous antibody response. Remarkably, in both groups, responses in persons with a history of vaccination were skewed towards older seasonal H1 antigens. These details were not evident from the kinetics in HI titer, the method routinely used for evaluation of antibody responses.

The study confirms that antibody response to infection and vaccination are both shaped by prior exposure history, where prior regular seasonal influenza virus vaccination had a clear effect on the magnitude and kinetics of response to pandemic influenza infection and vaccination. The higher GMTs at baseline were particularly clear for antibodies binding to HA1 peptide from recent seasonal influenza viruses, but slightly elevated levels were also observed for antigens not offered through vaccination within subtype H1. These reactivities may reflect the broadening of antibody response with age. [15] An intriguing question is whether such antibodies influence outcome of infection. Studies during the pandemic have been inconclusive in this respect: The presence of cross-neutralizing antibodies was limited to higher age groups, and no protective effect was expected or observed in several studies. [6], [18], [19] In contrast with this, some degree of cross protection from severe illness by prior seasonal vaccination was suggested in some studies. [20], [21], [22] Prior infection with an influenza A virus can reduce morbidity and mortality caused by an infection with an antigenically divergent influenza A virus because of heterosubtypic immunity, both within and between subtypes. [23] Both natural infection and seasonal vaccination can induce heterosubtypic neutralizing antibodies, but our data suggest skewing against such antibodies in persons with a history of seasonal vaccination. [8], [9], [10] The immunological basis of heterosubtypic immunity is not fully understood, but B cells, CD4^+^ and CD8^+^ T-cells and mucosal immunity may contribute. [11], [23] Passive serum transfer showed that antibodies induced by seasonal influenza A(H1N1) virus conferred protection in naïve recipient mice against A(H1N1)pdm09 challenge. The presence or absence of HI antibodies, therefore, is not the sole indicator of the effectiveness of protective cross-reactive antibody immunity. [24] In a mouse model, Hillaire et al. [25], demonstrated that induction of T cells specific for a seasonal H3N2 influenza virus led to protection against infection with the antigenically unrelated A(H1N1)pdm09. In addition, repeated infection with seasonal influenza virus improved protection and clearance of influenza A(H1N1)pdm09 in ferrets. [26] In young children, a difference was observed in levels of CD8+ T cells between vaccinated and unvaccinated individuals, suggesting that the same mechanisms may apply in humans as postulated by Bodewes et al. [27], [28] The influenza pandemic of 2009 showed an unbalanced age distribution of infected individuals, with a low incidence in elderly and a high incidence in children. This could be partly explained by the lack of heterosubtypic immunity, as a proportion of young children are immunologically naïve for influenza viruses. [6], [29] However, others described that low level heterosubtypic antibody responses following seasonal influenza vaccination could offer immune protection against antigenically distinct influenza viruses to a certain extent. [9], [10], [30] The observations above imply that seasonal influenza vaccines should also be evaluated for their capacity to mimic the balance in response triggered by wild type infection. Studies from Skowronski et al. [31], [32] suggested an increased risk of illness in persons with a history of seasonal influenza vaccination during the pandemic, which potentially could be explained by reduced levels of cross protective antibodies or T cells as a result of reduced wild type infection.

### Conclusions

In this study, we show that a history of seasonal influenza vaccination has different effects on infection and vaccination response. In vaccinees, the level of antibodies to the homologous strain was reduced in persons with a history of vaccination, whereas the reverse was true for infected persons. In both groups, however, the antibody response was skewed against heterologous antigens. More research is needed to understand if these observations are relevant for susceptibility of the individuals for infection and disease. We also conclude that improved assessment of the quality of immune response is needed when evaluation current and potential influenza vaccines.

## Supporting Information

Table S1 **Geometric mean titers (GMT) at baseline and after natural infection with pandemic influenza H1 2009.** GMT estimates are expressed as fold change for GMTs in persons with and without a history of seasonal vaccination (GMTvaccinated/GMTnonvaccinated).
(DOC)

Table S2 **Geometric mean titers (GMT) at baseline and after vaccination with pandemic H1 2009 vaccine of persons with and without a history of seasonal vaccination.**
(DOC)
