# Supplementary material for: Profiling of Humoral Response to Influenza A(H1N1)pdm09 Infection and Vaccination Measured by a Protein Microarray in Persons with and without History of Seasonal Vaccination
Source: PLoS One. 2013 Jan 24;8(1):e54890. doi: 10.1371/journal.pone.0054890 (PMC3554683; doi:10.1371/journal.pone.0054890)
Supplement: Table S1 — Geometric mean titers (GMT) at baseline and after natural infection with pandemic influenza H1 2009. GMT estimates are expressed as fold change for GMTs in persons with and without a history of seasonal vaccination (GMTvaccinated/GMTnonvaccinated). (DOC) [file pone.0054890.s001.doc]

**Table S3**. Geometric mean titers (GMT) at baseline and after natural infection with pandemic influenza H1 2009. GMT estimates are expressed as fold change for GMTs in persons with and without a history of seasonal vaccination (GMTvaccinated/GMTnonvaccinated).

|  | estimate | 95 % CI | | p-value |
| --- | --- | --- | --- | --- |
|  |  | lower | upper |  |
| **H1-1918** |  |  |  |  |
| GMT (time=0; no former seas. vac.) | 29.2 | 16.6 | 51.3 |  |
| Fold change at T = 1* | 2.95 | 1.98 | 4.40 | < 0.0005 |
| Fold change at T = 2* | 3.46 | 2.37 | 5.06 | < 0.0005 |
| Ratio former/no former seas. vac. | 2.49 | 1.12 | 5.58 | 0.027 |
| **H1-1933** |  |  |  |  |
| GMT (time=0; no former seas. vac.) | 37.8 | 21.1 | 67.4 |  |
| Fold change at T = 1* | 2.42 | 1.61 | 3.63 | < 0.0005 |
| Fold change at T = 2* | 2.76 | 1.91 | 4.00 | < 0.0005 |
| Ratio former/no former seas. vac. | 2.72 | 1.22 | 6.09 | 0.017 |
| **H1-1999** |  |  |  |  |
| GMT (time=0; no former seas. vac.) | 66.6 | 36.2 | 122.3 |  |
| Fold change at T = 1* | 1.92 | 1.29 | 2.85 | 0.002 |
| Fold change at T = 2* | 2.05 | 1.35 | 3.11 | 0.001 |
| Ratio former/no former seas. vac. | 5.77 | 2.47 | 13.45 | < 0.0005 |
| **H1-2007** |  |  |  |  |
| GMT (time=0; no former seas. vac.) | 73.4 | 37.3 | 144.3 |  |
| Fold change at T = 1* | 2.13 | 1.47 | 3.11 | < 0.0005 |
| Fold change at T = 2* | 2.46 | 1.66 | 3.66 | < 0.0005 |
| Ratio former/no former seas. vac. | 5.74 | 2.25 | 14.68 | 0.001 |
| **H1-2009** |  |  |  |  |
| GMT (time=0; no former seas. vac.) | 30.1 | 16.9 | 53.8 |  |
| Fold change at T = 1* | 3.74 | 2.30 | 6.09 | < 0.0005 |
| Fold change at T = 2* | 5.45 | 3.55 | 8.38 | < 0.0005 |
| Ratio former/no former seas. vac. | 2.23 | 0.99 | 5.03 | 0.053 |
| **H2-1957** |  |  |  |  |
| GMT (time=0; no former seas. vac.) | 32.4 | 21.8 | 48.2 |  |
| Fold change at T = 1* | 1.42 | 1.09 | 1.85 | 0.011 |
| Fold change at T = 2* | 1.55 | 1.21 | 1.98 | 0.001 |
| Ratio former/no former seas. vac. | 1.12 | 0.63 | 2.00 | 0.685 |
| **H3-2003** |  |  |  |  |
| GMT (time=0; no former seas. vac.) | 98.4 | 61.3 | 158.0 |  |
| Fold change at T = 1* | 1.37 | 1.12 | 1.67 | 0.004 |
| Fold change at T = 2* | 1.33 | 1.04 | 1.71 | 0.026 |
| Ratio former/no former seas. vac. | 2.47 | 1.24 | 4.94 | 0.012 |
| **H3-2007** |  |  |  |  |
| GMT (time=0; no former seas. vac.) | 105.1 | 68.6 | 161.0 |  |
| Fold change at T = 1* | 1.23 | 1.00 | 1.51 | 0.050 |
| Fold change at T = 2* | 1.39 | 1.13 | 1.71 | 0.003 |
| Ratio former/no former seas. vac. | 2.35 | 1.26 | 4.38 | 0.009 |
| **H5-2004** |  |  |  |  |
| GMT (time=0; no former seas. vac.) | 10.6 | 8.5 | 13.3 |  |
| Fold change at T = 1* | 1.11 | 0.97 | 1.27 | 0.135 |
| Fold change at T = 2* | 1.19 | 0.97 | 1.46 | 0.091 |
| Ratio former/no former seas. vac. | 1.06 | 0.77 | 1.44 | 0.723 |
| **H7-2003** |  |  |  |  |
| GMT (time=0; no former seas. vac.) | 12.5 | 10.0 | 15.7 |  |
| Fold change at T = 1* | 1.25 | 1.04 | 1.50 | 0.020 |
| Fold change at T = 2* | 1.36 | 1.11 | 1.67 | 0.004 |
| Ratio former/no former seas. vac. | 1.02 | 0.74 | 1.40 | 0.893 |
| **H9-1999** |  |  |  |  |
| GMT (time=0; no former seas. vac.) | 18.1 | 10.0 | 32.5 |  |
| Fold change at T = 1* | 1.66 | 1.25 | 2.21 | 0.001 |
| Fold change at T = 2* | 2.16 | 1.53 | 3.07 | < 0.0005 |
| Ratio former/no former seas. vac. | 1.44 | 0.61 | 3.38 | 0.395 |

Titers of antibodies to 7 different influenza HA1 antigens (left column) were measured by protein microarray. Table shows GMT at the first sampling date, fold changes at time points 1 and 2, and ratio between persons with and without a history of seasonal vaccination (estimates from linear mixed modeling, adjusted for gender and age).

* Fold change is calculated against T= 0 as a reference.
