# Supplementary material for: Profiling of Humoral Response to Influenza A(H1N1)pdm09 Infection and Vaccination Measured by a Protein Microarray in Persons with and without History of Seasonal Vaccination
Source: PLoS One. 2013 Jan 24;8(1):e54890. doi: 10.1371/journal.pone.0054890 (PMC3554683; doi:10.1371/journal.pone.0054890)
Supplement: Table S2 — Geometric mean titers (GMT) at baseline and after vaccination with pandemic H1 2009 vaccine of persons with and without a history of seasonal vaccination. (DOC) [file pone.0054890.s002.doc]

**Table S4.** Geometric mean titers (GMT) at baseline and after vaccination with pandemic H1 2009 vaccine of persons with and without a history of seasonal vaccination.

|  | No former seas. vac. | | | Former seas. vac. | | | Ratio former/no former seas. vac. | | | |
| --- | --- | --- | --- | --- | --- | --- | --- | --- | --- | --- |
|  | est. | 95 % CI | | est. | 95 % CI | | est. | 95 % CI | | p-value |
|  |  | lower | upper |  | lower | upper |  | lower | upper |  |
| **H1-1918** |  |  |  |  |  |  |  |  |  |  |
| GMT 0 | 29.4 | 21.5 | 40.2 | 51.6 | 35.4 | 75.3 | 1.76 | 1.07 | 2.89 | 0.027 |
| Fold change at T = 1* | 6.00 | 4.56 | 7.90 | 1.69 | 1.22 | 2.36 | 0.28 | 0.18 | 0.43 | < 0.0005 |
| Fold change at T = 2* | 4.44 | 3.22 | 6.11 | 2.03 | 1.39 | 2.97 | 0.46 | 0.28 | 0.75 | 0.002 |
| **H1-1933** |  |  |  |  |  |  |  |  |  |  |
| GMT 0 | 20.4 | 15.7 | 26.6 | 36.7 | 26.7 | 50.4 | 1.79 | 1.18 | 2.73 | 0.007 |
| Fold change at T = 1* | 2.89 | 2.34 | 3.57 | 1.41 | 1.09 | 1.82 | 0.49 | 0.35 | 0.68 | < 0.0005 |
| Fold change at T = 2* | 2.37 | 1.84 | 3.05 | 1.73 | 1.28 | 2.34 | 0.73 | 0.49 | 1.08 | 0.115 |
| **H1-1999** |  |  |  |  |  |  |  |  |  |  |
| GMT 0 | 27.4 | 20.3 | 37.0 | 220.6 | 153.2 | 317.7 | 8.05 | 4.98 | 13.01 | < 0.0005 |
| Fold change at T = 1* | 3.40 | 2.64 | 4.37 | 1.21 | 0.90 | 1.64 | 0.36 | 0.24 | 0.53 | < 0.0005 |
| Fold change at T = 2* | 3.02 | 2.32 | 3.94 | 1.25 | 0.91 | 1.71 | 0.41 | 0.27 | 0.62 | < 0.0005 |
| **H1-2007** |  |  |  |  |  |  |  |  |  |  |
| GMT 0 | 23.7 | 18.0 | 31.3 | 138.4 | 98.9 | 193.7 | 5.84 | 3.75 | 9.09 | < 0.0005 |
| Fold change at T = 1* | 3.63 | 2.90 | 4.54 | 1.34 | 1.03 | 1.76 | 0.37 | 0.26 | 0.53 | < 0.0005 |
| Fold change at T = 2* | 3.12 | 2.45 | 3.97 | 1.39 | 1.04 | 1.85 | 0.44 | 0.31 | 0.65 | < 0.0005 |
| **H1-2009** |  |  |  |  |  |  |  |  |  |  |
| GMT 0 | 25.3 | 18.9 | 33.8 | 37.9 | 26.6 | 53.8 | 1.50 | 0.95 | 2.38 | 0.085 |
| Fold change at T = 1* | 9.58 | 7.03 | 13.05 | 2.44 | 1.68 | 3.55 | 0.26 | 0.16 | 0.41 | < 0.0005 |
| Fold change at T = 2* | 8.54 | 6.03 | 12.11 | 3.65 | 2.41 | 5.53 | 0.43 | 0.25 | 0.73 | 0.002 |
| **H2-1957** |  |  |  |  |  |  |  |  |  |  |
| GMT 0 | 32.2 | 24.3 | 42.7 | 40.9 | 29.0 | 57.6 | 1.27 | 0.81 | 1.99 | 0.296 |
| Fold change at T = 1* | 1.67 | 1.41 | 1.98 | 1.11 | 0.90 | 1.35 | 0.66 | 0.51 | 0.86 | 0.003 |
| Fold change at T = 2* | 1.56 | 1.33 | 1.84 | 1.09 | 0.90 | 1.33 | 0.70 | 0.54 | 0.90 | 0.006 |
| **H3-2003** |  |  |  |  |  |  |  |  |  |  |
| GMT 0 | 64.7 | 51.2 | 81.8 | 134.5 | 101.3 | 178.7 | 2.08 | 1.43 | 3.02 | < 0.0005 |
| Fold change at T = 1* | 1.71 | 1.46 | 1.99 | 1.18 | 0.98 | 1.42 | 0.69 | 0.54 | 0.88 | 0.003 |
| Fold change at T = 2* | 1.38 | 1.16 | 1.64 | 1.22 | 0.99 | 1.50 | 0.88 | 0.68 | 1.15 | 0.360 |
| **H3-2007** |  |  |  |  |  |  |  |  |  |  |
| GMT 0 | 33.2 | 26.0 | 42.5 | 71.5 | 53.1 | 96.3 | 2.15 | 1.45 | 3.19 | < 0.0005 |
| Fold change at T = 1* | 1.14 | 1.05 | 1.25 | 0.97 | 0.87 | 1.08 | 0.84 | 0.73 | 0.97 | 0.020 |
| Fold change at T = 2* | 1.00 | 0.88 | 1.13 | 1.03 | 0.89 | 1.20 | 1.03 | 0.85 | 1.25 | 0.723 |
| **H5-2004** |  |  |  |  |  |  |  |  |  |  |
| GMT 0 | 10.0 | 9.6 | 10.5 | 10.0 | 9.5 | 10.5 | 1.00 | 0.93 | 1.07 | 0.892 |
| Fold change at T = 1* | 1.05 | 1.00 | 1.10 | 1.00 | 0.94 | 1.06 | 0.95 | 0.88 | 1.02 | 0.166 |
| Fold change at T = 2* | 1.03 | 0.98 | 1.08 | 1.00 | 0.94 | 1.06 | 0.97 | 0.90 | 1.04 | 0.404 |
| **H7-2003** |  |  |  |  |  |  |  |  |  |  |
| GMT 0 | 10.8 | 9.7 | 12.0 | 12.6 | 11.0 | 14.4 | 1.17 | 0.98 | 1.39 | 0.081 |
| Fold change at T = 1* | 1.06 | 0.98 | 1.15 | 1.06 | 0.97 | 1.17 | 1.00 | 0.89 | 1.13 | 0.991 |
| Fold change at T = 2* | 1.08 | 0.97 | 1.20 | 1.12 | 0.99 | 1.27 | 1.03 | 0.88 | 1.22 | 0.701 |
| **H9-1999** |  |  |  |  |  |  |  |  |  |  |
| GMT 0 | 11.5 | 10.0 | 13.2 | 14.2 | 12.0 | 16.8 | 1.24 | 0.99 | 1.55 | 0.060 |
| Fold change at T = 1* | 1.57 | 1.34 | 1.83 | 1.05 | 0.87 | 1.27 | 0.67 | 0.53 | 0.86 | 0.002 |
| Fold change at T = 2* | 1.48 | 1.26 | 1.74 | 1.20 | 0.99 | 1.45 | 0.81 | 0.63 | 1.04 | 0.101 |

Titers of antibodies to 7 different influenza HA1 antigens (left column) were measured by protein microarray. Table shows GMT at the first sampling date, and fold changes at time points 1 and 2 and ratio between persons with and without a history of seasonal vaccination (estimates from linear mixed modeling, adjusted for gender and age).
* fold change is calculated against T= 0 as a reference.
